# HLA-Epitope Matching or Eplet Risk Stratification: The Devil Is in the Details

**DOI:** 10.3389/fimmu.2018.02010

**Published:** 2018-08-31

**Authors:** Anat R. Tambur

**Affiliations:** Comprehensive Transplant Center, Feinberg School of Medicine, Northwestern University, Chicago, IL, United States

**Keywords:** eplet, epitope, HLA, matching risk stratification, transplantation, compatibility

## Abstract

“Epitope matching” became a trending topic in organ transplantation. In fact, discussions on clinical implementation and utilization of this approach in organ allocation algorithms are currently on-going. More recently, the term “eplet mismatch load” was introduced in publications. While the terms are often used synonymously, they are NOT equivalent. This short overview is meant to emphasize the differences between the terms epitope matching and eplet mismatching (or mismatch load) as well as to provide perspective on different approaches for interpretation of immune compatibility between the donor of an organ transplant and the recipient. It highlights some of the less explored qualities of HLA-epitopes, and stresses the need to understand the differences between donor and recipient in terms of immunogenicity and ability to initiate an immune response. While the field of “epitope matching” shows enormous promise, it is still in its infancy. What is sorely missing is understanding of EPITOPE COMPATIBILITY rather than matching. Further work is required before new approaches can be introduced into routine clinical practice and organ allocation schemes.

“Epitope matching” became a trending topic in organ transplantation. A number of recent publications initiated debates whether “epitope matching” should replace antigen matching. HLA laboratories are receiving requests from physicians to perform “epitope matching,” and there are even suggestions to implement “epitope matching” in UNOS organ allocation algorithm.

To avoid putting the cart in front of the horse, it is important to define the meaning, appreciate nuances in terminology, and understand the exact purpose of such approach. At this point, the number of open questions is higher than the number of definitive answers: How are we defining “epitopes”? Is it simply differences in amino-acid sequences? Are we talking about “eplets” rather than “epitopes”? Is that only semantics or does each term represent different entities with different effects on the immune response? Importantly, can we assume that each amino-acid change affects immune reactivity to the same degree? How would we quantify a change between 2 amino-acid with similar properties (e.g., both are non-polar and small) vs. a change between 2 amino-acid with divergent properties (one is small, non-polar; and the other is bulky and positively charged)? Logistically speaking, should “epitope matching” be applied pre-transplantation for allocation purposes; or should it be used at the post-transplant period? If the former—what algorithm should be used? Are we to give the same weight to matching at class I and class II eplets? Should the weight of HLA-DQ be higher knowing it is the more common culprit in poor graft outcome? How would changes in algorithm affect organ allocation and equity for the different ethnic groups? Will it inflate or deflate known differences? To address these questions one need to first gain understanding to the concept of epitopes in HLA.

## Understanding epitopes

The past decade witnessed a burst in the use of the term HLA-epitopes in transplant related literature. Interestingly, there was a similar peak, albeit much shorter, around 1976–1978. This is an important observation as the concept of HLA antibodies recognizing a specific HLA epitope rather than the whole HLA antigen was already appreciated in the early days of histocompatibility testing (1–3). At the time, though, the term “epitope” was used in a vague sense, i.e., describing serologic reactivity patterns. As an example, 3 different serum samples can each identify one of the following 3 HLA antigens—A2, A68, B57. A 4th serum can react positively with both A2 and A68, but not B57; another serum can react with A2 and B57, but not A68; and yet another serum can react with all 3 antigens. This observation was explained by postulating an area of the HLA antigen (termed “epitope”) that is unique to each of the A2, A68, and B57 antigens, concomitant with a different target within A2 and A68 (but not present in B57) to which the 4th serum binds. The same rationale applies for A2 and B57, and A2/A68/B57. Thus, A2 and A68 share an epitope—a target for antibody recognition, different from the epitope shared by A2 and B57, and yet another epitope is shared by A2, A68, and B57. All these are in addition to the epitopes that are unique to the individual antigens.

**Figure 1:**
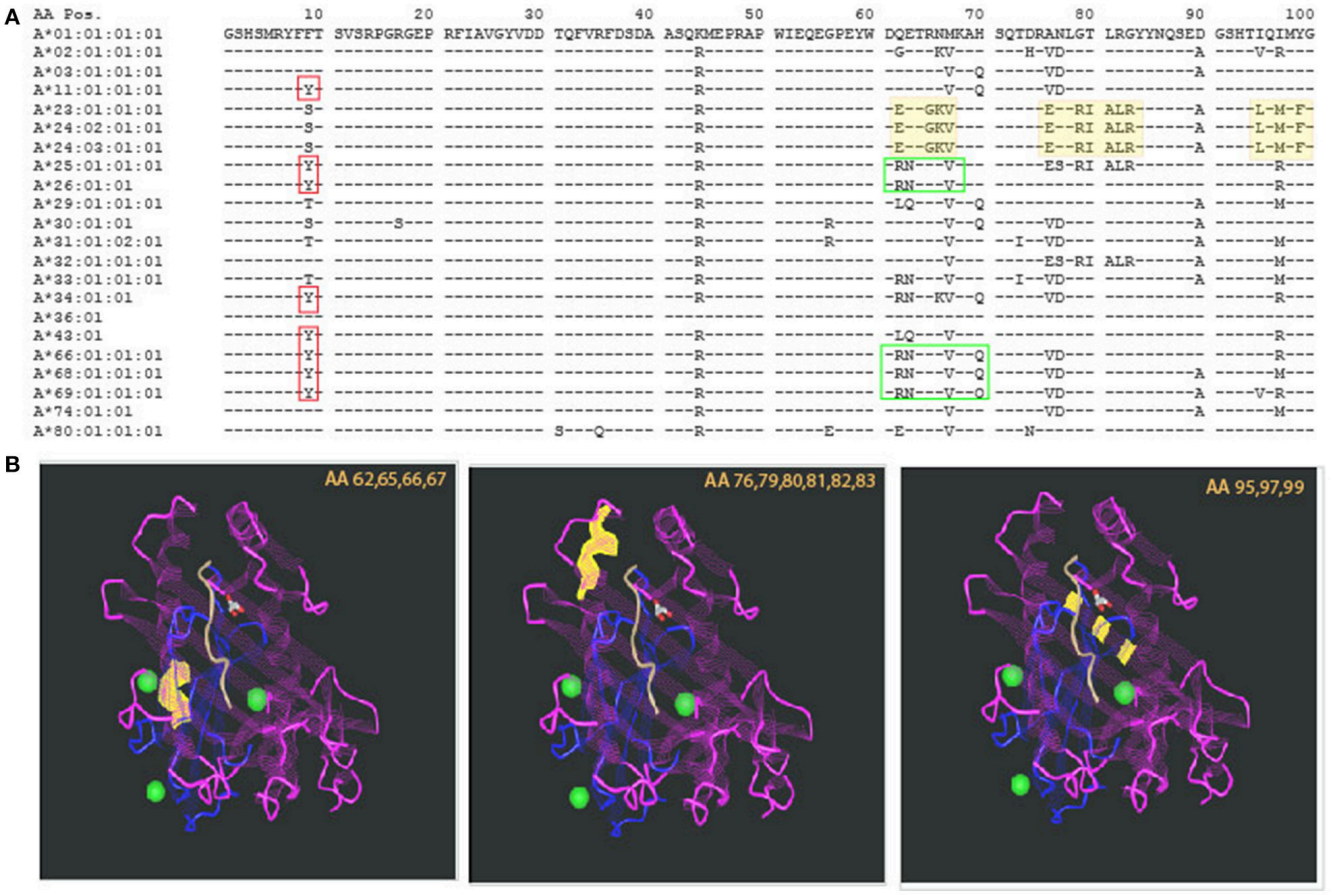
Polymorphism and homology in the HLA system. **(A)** The sequence of the first 100 amino acids of HLA-A alleles is presented using the one letter naming convention. Allele identity is listed to the left. Consensus sequence is represented by the sequence of HLA-A*01:01:01:01. For additional sequences, homology to the consensus sequence is illustrated by a dash sign (“−”). The vast majority of the other alleles' sequences are homologous to the consensus sequence. Polymorphism is represented by a single letter designation of the different amino acid. Even among polymorphic positions, there is identity with some of the other alleles. This is demonstrated by the red boxes—all of those alleles have Y in position 9 but other alleles are identical to the consensus sequence (F) and yet others have (S). Other examples are illustrated by the green boxes. **(B)** Polymorphism can be distributed in different areas of the HLA molecule. Three examples are illustrated by the yellow highlights in the sequence, and the corresponding sites of the molecule are shown in the 3 insets, listing the polymorphic amino acids that are highlighted in yellow. Those are located, from left to right: at the lower edge of the alpha helix, around center molecule; the alpha helix at the edge of the peptide binding groove; and at the bottom of the peptide binding groove—beta pleated sheets. The projected effects on T cell receptor and the bound peptide are likely to change based on the location of the polymorphism.

The introduction of molecular typing technique and elucidation of the 3D structure of an HLA molecule helped in deciphering those intricate antibody recognition patterns. As the amino acid [AA] sequence of HLA antigens unraveled, a unique property of the HLA system emerged. While the HLA system is the most polymorphic system known, it also has a very high degree of homology. This description may sound like an oxymoron, but at the population level, it is required in order to support the physiologic role of the HLA system, namely, presentation of wide range of foreign proteins (viruses, bacteria, etc.) and activation of immune response (4). The concept of high polymorphism in the context of high homology is illustrated in Figure 1A, showing AA residues coding for common HLA-A antigens. The top sequence, HLA-A^*^01:01:01:01, is referred to as the consensus sequence; each of the dashes (“−”) in following sequences represent identity to the consensus sequence at that particular location. The high level of homology is evident by the high prevalence of “−.” Polymorphism is indicated by the presence of an AA different from the consensus sequence (using single letter designation). Importantly, some of the polymorphic regions appear in several antigens, making those AA both homologous and polymorphic dependent on the sequence to which they are compared with (red and green boxed areas, Figure 1A). Comparing AA sequences to antibody reactivity patterns revealed some correlation between the two, leading to the supposition that there is an association between AA sequence similarities or differences and antibody reactivity. In fact, this is the basis of the HLA-matchmaker software developed by Rene Duquesnoy, using triplets and eplets to describe amino acids in linear and non-contiguous sequences.

## Using eplet mismatch load information for clinical purposes

Earlier reports using HLAMatchmaker provided insight into how one can interpret antibody reactivity patterns. For example—providing rationale to common observations of patients, exposed to a particular donor HLA-mismatch, developing antibodies not only against this donor but also against additional (non-donor) HLA antigens (5–7); explanation for development of antibodies only to a subset of alleles within a single antigen (at the serological level) but not to other alleles within that antigen family of alleles (8, 9); etc.

The excitement that followed Duquesnoy's innovative software was quickly translated into a search for clinical applications. The term “Epitope Matching” followed quickly thereafter. However, when one delves into the details of published reports it becomes clear that less than a handful studies attempted to use “eplet matching” in a *prospective* manner, in order to *impact organ allocation* decisions. The vast majority of the published work utilized HLAMatchmaker in a retrospective fashion, to document that recipients of high eplet mismatch load organs had worse outcome.

Wiebe et al. (10) retrospectively analyzed the burden of HLA-class II eplet mismatch (MM) in 286 donor/recipient pairs demonstrating that patients with lower HLA-DR (<10) and patients with lower HLA-DQ (<17) eplet MM load, had significantly longer *de-novo* (dn)DSA free survival. Sapir-Pichhadze et al. (11) showed in a retrospective nested case-controlled study of 52 patients that patients with increased eplet MM load had higher transplant glomerulopathy rates (combined HLA-DR and -DQ MM load >27, and >43). Sullivan et al. (12) embarked on an enormous undertaking, analyzing eplet MM load in 4851 pediatric heart recipients transplanted between 1987 and 2012. Unfortunately, since HLAMatchmaker requires high resolution typing information, and the data in the SRTR is only at the serologic level, associations were found only between recipients with 2–4 class I mismatches and graft loss. An association between increased eplet MM load and Chronic Lung Allograft Dysfunction was reported by Walton et al. (13) using a cohort of 175 lung transplant recipients. The threshold used in this study was >48 HLA-DRB1/3/4/5 and HLA-DQA/B eplets MMs.

Wiebe et al. (14) added an extra layer combining the effect of increased eplet MM load with reduced trough levels of immunosuppression. Their cohort of 195 patients was previously analyzed for the effect of medication non-adherence, using electric monitors in medication vial caps. Patients with high MM load (>10 and >17 for HLA-DR and –DQ) that exhibited also immunosuppression non-adherence had decreased allograft survival over a-8-year follow-up period. A follow up study demonstrated correlation also between low trough levels of Tacrolimus in the presence of high alloimmune risk (threshold of >11 for either HLA-DR or –DQ eplet MM). Wiebe and colleagues concluded that not only that HLA-DR/DQ eplet MM and tacrolimus trough levels are independent predictors of dnDSA development, but importantly, recipients with high HLA alloimmune risk should not be target tacrolimus levels <5 hg/ml unless essential (15). The above studies strongly support the conclusion that, at the population level, patients with higher eplet MM load are more likely to have worse graft outcome. Moreover, these studies suggest that eplet MM load can provide guidance for risk stratification post transplantation, when considering immunosuppressive drug minimization strategies.

However, at the individual patient level, only one group attempted to use eplet MM load strategies to inform allocation. Kausman et al. (16) integrated donor eplet MM load into the kidney-donor acceptance criteria for deceased-donor-waitlist pediatric patients. The threshold for class I antigens was <10, and for class II <30 MM eplets. Nineteen patients were transplanted during the 1-year study period. Of those, 8 were chosen for the modified deceased-donor exclusion criteria; 8 received a living-donor transplant, with no exclusion; and 3 received deceased-donor transplant with no exclusion. It is important to note that the class II MM load was similar in the first 2 groups—about 20 class II eplet MM each whereas the latter group had over 60 MM. Development of *de-novo* DSA at the end of 1 year follow up for all patients was compared between the 3 groups revealing that 6/8 patients in the study group remained DSA negative compared with 2/3 and 3/8 of the patients with no eplet MM exclusion groups. A similar approach was utilized by this group to transplant 7 pediatric patients through the Australian Kidney Exchange (AKX) program (17) as well as in their adult Kidney Paired Exchange program (18). While potentially promising, this single center study includes very small cohorts, which precludes attaining conclusive results.

## Exploring other approaches beyond conventional HLA typing

A different approach, restricted to the very highly sensitized patients (>85% PRA), has been in use by Eurotransplant for over 25 years. In this scheme, HLAMatchmaker is used prospectively to identify all HLA antigens that the recipient does not possess antibodies against (current as well as historical; defined as Acceptable Mismatches—Acceptable MM). This selection is based on identification of all mismatched eplets that are present in the antigens targeted by the patients' serum but absent from their own HLA antigens. The universe of these mismatched eplets is then compared against the rest of the HLA specificities and any HLA antigen that carries one of those mismatched eplets is removed as well. Thus, two categories of HLA antigens are removed from the potential acceptable mismatched antigens: those that the recipient has proven antibodies to and those that share AA sequences with the first category, but no antibodies were reported. After excluding all of these antigens, the patient is left with a cohort of potentially acceptable antigens that includes his own HLA antigens and the antigens left after the exclusion (19). Of note, patients in the Acceptable MM program are offered donors from all geographic areas covered by Eurotransplant, as long as they fulfill one of two criteria. The donor and potential recipient have to be HLA-DR matched, or, they have to share one HLA-B and one HLA-DR antigens (at the serological level) (19). A recent report summarizing the Acceptable MM program with 10 years follow-up showed that these patients have significantly superior 10-year graft survival compared to highly sensitized patients transplanted on the basis of avoidance of unacceptable mismatches (20).

The concept of the acceptable MM program has been in use by Eurotransplant for about 25 years. While very successful, it is important to appreciate that it tackles a different aspect of matching, namely, extending the universe of unacceptable mismatches beyond those that the patients exhibit antibodies to. The immunological rationale driving this approach is that the patient is more likely to develop antibodies [or harbor memory (21)] to these additional antigens, and less likely to develop antibodies against HLA antigens that do not share mismatches with current antibody targets. This rationale is supported by multiple studies using the HLAMatchmaker software to explain the generation of 3rd party antibodies together with the generation of DSA (5–9).

## Fundamental insight into immunogenicity and antigenicity

The 3-dimentional structure of an HLA molecule has evolved during evolution to fit its role in presentation of foreign molecules to the cellular arm of the immune system. Specifically, the two most distal domains of an HLA molecule form a peptide-binding groove in which the foreign peptide is to be nestled (22). Accordingly, the polymorphic amino acids of an HLA molecule are concentrated in these two domains, mostly in areas that participate in forming the peptide-binding site and T cell receptor recognition site (23). Thus, it is important to appreciate that not every polymorphism will have the same impact on immune recognition. This is illustrated in Figure 1B, highlighting the location of the polymorphic regions shaded yellow in Figure 1A. The first polymorphism—amino acid (AA) positions 62,65,66,67 highlighted in yellow is located within the alpha helix, around the center of the molecule; the second—AA positions 76,79,80,81,82,83 is also located within the alpha helix but closer to the area that frames the edges of the peptide binding group; both of these polymorphic sites are likely to be part of both the peptide binding site as well as the TCR recognition site; lastly, the third polymorphism—AA positions 95,97,99 are actually buried below the peptide, within the beta pleated sheets, not directly accessible to TCR recognition, but definitely affecting the peptide that can be bound by the HLA molecule. While the exact impact of these polymorphisms has not been elucidated, it is expected that they will have different effects on immune reactivity. In fact, some evidence to this statement was already demonstrated by Paul Terasaki's group analyzing HLA antibody profiles of allosera and verification by absorption elution studies, leading to the Ter-Ep nomenclature (24). Not all the eplets identified by HLAMatchmaker have a Ter-Ep equivalent. Similarly, it is important to appreciate that only a few the eplets are considered verified in the epitope registry (25). This fact strongly suggests that not all computer predicted “epitopes” are associated with pathogenic responses.

**Figure 2:**
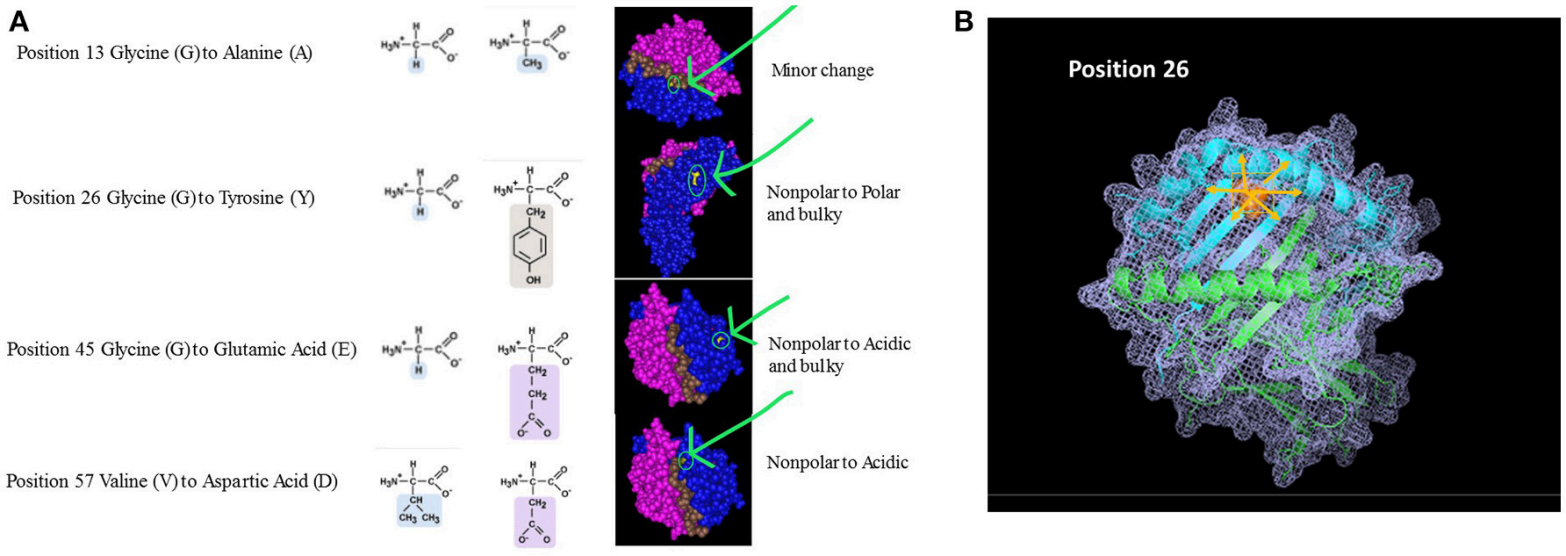
**(A)** Amino Acid substitutions can come in different flavors. Amino acids can be classified based on several characteristics: Polarity, Electrostatic charge, Aliphatic, Aromatic, Size, etc. The nature of the substitution and potentially its immunological magnitude is likely to be influenced by how similar or different the mismatched amino acid is. The examples given in this figure are taken from comparison between HLA-DQB1 sequences. The panels on the right highlight the area of the molecule where the substitution takes place, emphasizing the nature of the substitution based on the parameters listed above. **(B)** Ripple effect of an amino acid substitution on overall 3D structure. The single amino acid substitution in this example is at position 26, demonstrating an eplet mismatch between Glycine and Tyrosine. Tyrosine is polar and significantly bulkier than the small, non-polar Glycine (illustrated as small inserts at top right). Replacing Glycine with Tyrosine is likely to displace not only the amino acids adjacent to it in the sequence (2D; positions 25 and 27) but also all other neighboring amino acids at the 3D structure—shown in pink arrows.

Beyond the exact location of the mismatch, one should also consider the unique properties of the polymorphic amino acids (e.g., size, solubility, ionization properties). The polarity and charge of the amino acid can have a profound effect not only at the specific area of polymorphism but it can also affect neighboring AAs and thus impacting the structure and biological activity of the HLA molecule at large. Figure 2A shows 4 amino acid differences between HLA-DQ molecules. Assuming the recipient has an HLA-DQ with Glycine at position *13* and his donor has Alanine at that position, the substitution involves two AAs with similar characteristics. Moreover, position *13* is located at the bottom of the peptide-binding grove. The overall effect on the properties of the HLA molecule are therefore not likely to be significant, thus having low impact on initiating a significant alloimmune response. On the other hand, when the AA mismatch involves a substitution of a small nonpolar Glycine with a bulky and polar Tyrosine (position *26*), the expected effect is significantly higher. This substitution is located within the beta pleated sheets, just below the alpha helix framing the peptide-binding site. The mismatch between the recipient's Glycine at position *45* with a donor's Glutamic acid will introduce an acidic and bulky AA instead of the small nonpolar original component. This substitution is located further from the peptide binding site. Lastly, a similar scenario is illustrated by a mismatch between Valine and Aspartic acid at position *57*. In this example, the substitution is located within the alpha helix. The right-hand panel illustrates the location of these AA within the 3D structure of an HLA-DQ molecule. This view, however, does not indicate the extent of cascading ramification on the HLA-peptide-TCR or HLA-peptide-antibody interactions. In other words, these substitutions are likely to carry over to affect the location of neighboring AA, as they are bound to displace them even if no additional substitutions are present. One may expect that this change will be associated with a significant effect on allorecognition. Figure 2B displays one of these mismatches (position 26) using a stick diagram of an HLA-DQ molecule. Replacing Glycine with the much bulkier and polar Tyrosine is likely to displace not only the amino acids in position 25 and 27 that are neighboring in the 2D sequence, but also other amino acids that are adjacent to it in the 3D structure, including most likely AA 70–78. Thus, a change of one AA can drive a significant change of the dimensions HLA molecule as well as the peptides it can bind and its interactions with TCR and antibodies.

In fact, there are numerous examples in which the donor and recipient differ only by a single amino-acid difference and yet the recipient developed *de-novo* DSA. For example, HLA-A^*^02:01 and HLA-A02:06 differ by only one amino acid in position 9 (Phenylalanine to Tyrosine); HLA-DRB1^*^04:03 differs from DRB1^*^04:06 only in position 37 (Serine to Tyrosine); HLA-B^*^51:01 differs from HLA-B^*^52:01 only in position 62 (Glutamic acid to Asparagine), etc. Even more concerning is the observation that patients typed as HLA-DRB1^*^14:01 can exhibit antibodies against DRB1^*^14:06 and vice versa, although the one amino acid MM between these alleles is considered to be conservative (Glycine to Valine in position 86). The above examples clearly demonstrate that the *immunogenicity* of a mismatch between donor/recipient pair is a consequence of several factors and not just the number of amino acid or eplet mismatches.

Of note, additional approaches and tools are currently used to explore the concepts of immunogenicity and antigenicity, and whether we can apply those for better donor-recipient matching. Vasilis Kosmaliaptsis and his group are studying physiochemical disparities between donor and recipient (26, 27). Spierings and colleagues reason that since B cell responses are guided by T cell help, we should investigate PIRCHE-II [predicted indirectly recognizable HLA epitopes; (28, 29)]. Interestingly though, in a recent collaboration between the Manitoba and the Cambridge groups (30) comparing 3 approaches (donor HLA amino acid mismatch, electrostatic mismatch, and eplet mismatch), they concluded, “This report highlights that the use of one method over the other is likely to be driven by familiarity and ease of use as highly correlated results are produced by each method.” Similarly, the correlation between HLAMatchmaker or PIRCHE-II score and the incidence of *De-Novo* DSA following renal transplantation was virtually identical (31).

## In summary

- Eplet mismatch load analysis or Molecular Mismatching (32) is the best tool currently available for risk stratification at the population level.
- Implementation of an Acceptable MM program has been proven beneficial for organ allocation for the very highly sensitized patients.

HOWEVER:

- The difference between epitope and eplet must be better understood to help drive scientific advancement. This appreciation is critical in order to examine immunogenicity of different epitope incompatibilities.
- The consequences of different eplet (or amino acid) mismatches should be better understood—as shown above, not every mismatch will lead to the same type of immune response. Thus, two donor-recipient combinations that have an identical eplet MM load may exhibit very different immunogenicity schemes.
- While it is anticipated that epitope analysis be beneficial for all types of transplanted organs, its contribution and role in regulating immune responses for the different organs is not thoroughly investigated.

IMPORTANTLY:

- Currently there is little data to support the introduction of eplet analysis into allocation consideration and the push for introducing “epitope matching,” while appealing, is premature.
- Appreciation of EPITOPE COMPATIBILITY (or incompatibility) as modulator of immune responses is critical to guide equitable allocation schemes.

A word of caution—different permutations of eplet/epitope analysis software are currently in use. Validation of the software to be implemented by the allocation agencies, and a thorough comparison of software used by HLA laboratories is critical.

## Author contributions

The author confirms being the sole contributor of this work and approved it for publication.

### Conflict of interest statement

The author declares that the research was conducted in the absence of any commercial or financial relationships that could be construed as a potential conflict of interest.

## Abbreviations

AA: Amino Acids
MM: Mismatch
SRTR: Scientific Registry of Transplant Recipients.
